# A Disconnect Between Operational Evaluations of Larvicide Treatments to Storm Sewer Catch Basins and Findings of Larvicide Resistance in the Northwest Suburbs of Chicago, IL, USA

**DOI:** 10.3390/pathogens15090970

**Published:** 2026-09-11

**Authors:** Justin E. Harbison

**Affiliations:** 1 Department of Public Health Sciences, Loyola University Chicago, Maywood, IL 60153, USA; jharbison@luc.edu; 2 Northwest Mosquito Abatement District, Wheeling, IL 60090, USA

**Keywords:** catch basin, *Culex pipiens*, methoprene, mosquito, resistance

## Abstract

Regular evaluations of larvicide resistance and operational control effectiveness are two basic components of the Integrated Mosquito Management approach. It is therefore important for mosquito control programs to assess both the efficacy and the effectiveness of larvicide treatments. Recent observations in 2023 and 2024 within areas served by the Northwest Mosquito Abatement District (NWMAD) indicated that methoprene resistance categorized as high and extreme exist in populations of *Culex pipiens* (L). Despite more than 20 years of exclusive use of Altosid^®^ XR (2.1% methoprene) larvicide in NWMAD catch basins and associated expectations of reduced effectiveness, NWMAD’s evaluations of Altosid^®^ XR-treated catch basins in 2022, 2023, and 2025 found levels of control (emergence inhibition [EI]) to be similar to those reported in studies over the past 30 years of the same or similar larvicide formulation. An extreme abundance of caution is needed when interpreting findings of NWMAD’s control evaluations as these observational findings were collected over different years and areas served by NWMAD. These findings also lack inferential statistical analyses and were not collected in a scientifically rigorous and systematic manner with a true experimental design. The reported results should not be interpreted as demonstrating that methoprene resistance does not affect operational control.

## 1. Introduction

For the four mosquito abatement districts that serve the Chicago, IL, USA metropolitan area, larvicide applications to storm sewer catch basins are a primary part of mosquito control operations [1]. These catch basin treatments are performed to reduce populations of West Nile virus vectors, particularly *Culex pipiens* Linnaeus, 1758 [1]. Prior to 2025, one of these abatement districts, the Northwest Mosquito Abatement District (NWMAD), exclusively applied Altosid^®^ XR extended-release briquets (2.1% methoprene: Wellmark International, Schaumburg, IL, USA) to roughly 50,000 catch basins within approximately 270 sq mi (699.3 sq km) of the northwest suburbs of Chicago for more than 20 consecutive years [2].

According to the United States Centers for Disease Control and Prevention (CDC): “For vector control to be effective, mosquitoes must be susceptible to the insecticide selected for use.” [3]. Therefore, routinely testing for larvicide resistance is considered critical in the control of disease and pest mosquito species and one of the basic components of Integrated Mosquito Management (IMM) [4]. Over 2023 and 2024, larvicide resistance to methoprene was assessed in *Cx. pipiens* collected from 16 sites within the area served by NWMAD [2,5]. In these two studies, resistance was measured using bioassays that compare mortality of *Cx. pipiens* larvae from field-collected egg rafts to *Cx. pipiens* larvae from a laboratory susceptible strain with increasing doses of methoprene. Field egg rafts were collected from one to four aboveground gravid ovitraps baited with an alfalfa pellet infusion at each of NWMAD’s 16 sites. More detailed descriptions of the larvicide resistance bioassay methods and sites utilized in the Chicago metropolitan area are reported elsewhere [2,5,6].

**Figure 1:**
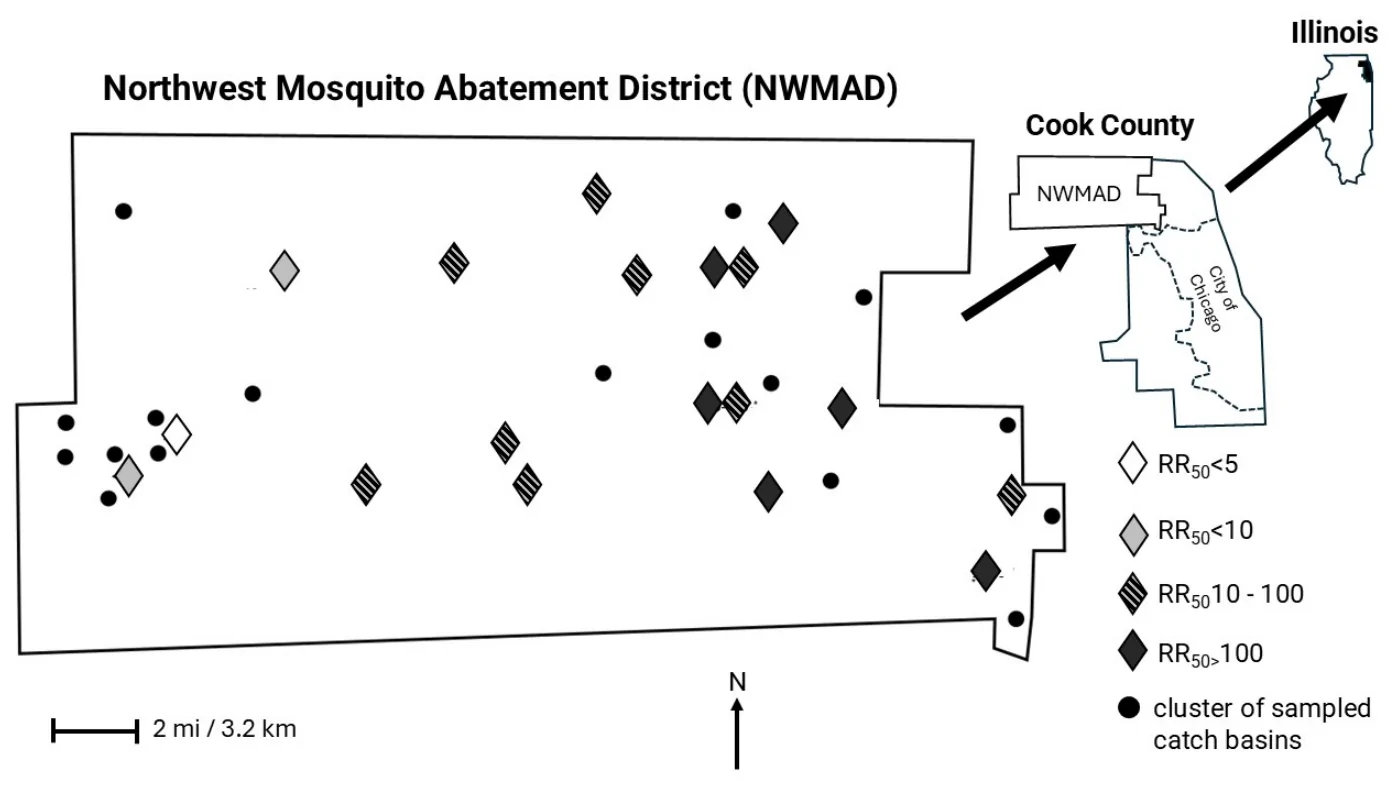
Approximate locations of clusters of up to 20 sampled basins and sites where LC_50_ resistance ratios (RR_50_) observed for *Culex pipiens* (L.) within the area served by the Northwest Mosquito Abatement District from 2022–2024. Locations of resistance sites and categories of RR_50_ are adapted from previously reported findings [2,5].

Briefly, from these bioassays, resistance ratios (RR_50_) were calculated as the estimated lethal concentration of active ingredient needed to kill 50% of larvae from field-collected egg rafts (LC_50_) divided by the LC_50_ of the susceptible lab strain. Mortality was assessed by counting dead larvae, pupae, and adults that failed to completely emerge. Resistance ratios (RR_50_) were then used to estimate how much more of an active ingredient is needed to kill wild populations in comparison to laboratory susceptible strains. For example, a RR_50_ of 10 suggests that 10 times the active ingredient would be needed to kill wild populations than the lab strain. Results from this work indicated that RR_50_ values characterized as “high” and “extreme” (RR_50_ > 10 or >100) were identified in *Cx. pipiens* populations in the eastern two-thirds of NWMAD and RR_50_ values characterized as “susceptible” to “moderate resistance” (RR_50_ < 5 or <10) observed in populations of that species in the western third of the district (Figure 1). In Lopez et al. [5], the RR_50_ values to methoprene were reported as ranges only (<10, 10–100, and >100). The referenced RR_50_ categories, RR < 5 = “susceptible”, 5–10 = “moderate resistance”, and >10 = “high”, are from the WHO guidance and were created for evaluation in *Aedes* genera [7]. Lopez et al. [2] created an additional category for *Cx. pipiens*: RR > 100 = extreme resistance. Implicit with the reporting of these categories is the assumption that these inform the degree to which larvicides associated with tested active ingredients can be less or not at all effective.

Based on these levels of reported resistance, as defined by the WHO [7] and Lopez et al. [2], there were concerns of reduced control with methoprene-based larvicides. Therefore, in 2025, NWMAD leadership (including the author, a NWMAD trustee) chose to switch out Altosid^®^ XR for two new non-methoprene larvicides in areas where high and extreme methoprene resistance was observed in *Cx. pipiens*. Basins in these areas (the eastern two-thirds of the district) received either one Natular^®^ XRT extended-release tablet (spinosad: Clarke, Roselle, IL, USA) or three water soluble pouches (WSPs) of Sumilarv^®^ 0.5G (pyriproxyfen: MGK, Minneapolis, MN, USA, 75 g). Catch basins in the remaining western third of the district continued to receive Altosid^®^ XR briquets where less methoprene resistance or susceptibility was observed.

## 2. Materials and Methods

Like resistance testing, monitoring the effectiveness of control efforts is another basic component of IMM as reported by the CDC [4]. Therefore, NWMAD routinely samples larvicide-treated basins to assess how well currently used larvicides are working and to evaluate new ones. From these efforts, there was an opportunity to assess the effectiveness of Altosid^®^ XR briquets in areas with populations of *Cx. pipiens* with high and extreme resistance and in areas where populations were believed to harbor more susceptibility to methoprene. In 2022 and 2023, clusters of up to 20 Altosid^®^ XR-treated basins were sampled to assess larvicide effectiveness in an area where high and extreme resistance was found in *Cx. pipiens* in 2023 and 2024. Later, in 2025, clusters of up to 20 Altosid^®^ XR-treated basins located in areas where it was believed more methoprene-susceptible *Cx. pipiens* populations exist were also sampled in the same manner. Basins within each cluster were located within an approximately 1 sq mi/2.6 sq km of each other. All sampling was performed by the author as part of NWMAD’s regular control evaluations. Clusters of sampled basins in the high/extreme resistance areas of NWMAD were approximately 5 mi (8 km) apart from those in moderate/susceptible areas. Sampling of all basins occurred by taking 5 dips of a standard 350 mL dipper with approximately 1 min given in between dips. Convenience sampling was used to find basins with safe access that minimized proximity to traffic. All pupae observed from a sampled basin were counted and placed in a Dart™ Solo UltraClear (Dart Container Corporation, Mason, MI, USA) 16 oz. clear polyethylene terephthalate Plastic Squat Cold Cup along with water from the catch basin and then covered with a Dart™ Solo Clear Flat Lid with Straw Slot Cup (Dart Container Corporation, Mason, MI, USA). Cups were monitored daily, and adults that successfully emerged were recorded. The percent control (estimated emergence inhibition) was calculated using the number of adults that did not successfully emerge from pupae in each cup. While untreated basins were not sampled as the NWMAD policy is to treat all basins that resources allow, prior work using similar methods suggests that emergence in untreated samples is >90% [8]. During all years, basins were sampled every 1 to 3 weeks through 16 weeks of treatment. Over 2022 and 2023, pupae were collected from a total of 226 unique sampled basins and in 2025, pupae were collected from 90 unique basins sampled.

## 3. Results

**Figure 2:**
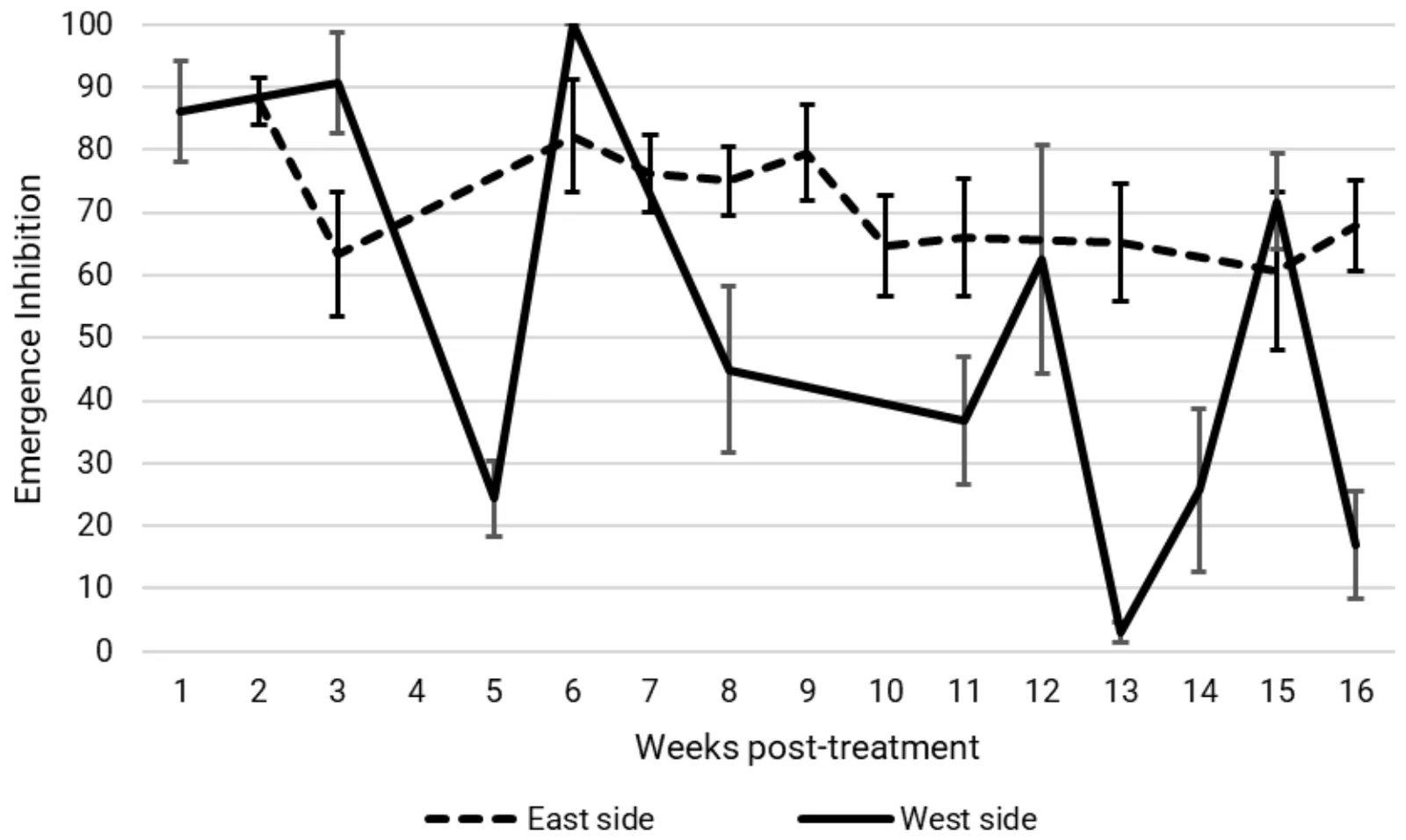
Percent emergence inhibition ± SE observed from catch basins treated with an Altosid^®^ XR briquet that were sampled in 2022 and 2023 in the east side of the Northwest Mosquito Abatement District, IL (methoprene resistance categorized as high and extreme) and in 2025 in the west side of the District (methoprene resistance categorized as susceptible to moderate).

Generally, Altosid^®^ XR-treated basins in the more methoprene-susceptible western area had lower observed control (average over 16 weeks = 47.9% SE ± 4.5%, N = 90) than those in the eastern area where high and extreme resistance was observed (average control over 16 weeks = 74.0% ± 2.2, N = 226) (Figure 2). Additionally, at 16 weeks post-treatment, the percent control in the more methoprene-susceptible area was 17.0% ± 8.5, N = 5 and 67.9% ± 7.3, N = 18 for the more resistant area. These results were surprising given the expectation that control would be reduced in areas associated with high and extreme methoprene resistance. Interestingly, the control observed in the high and extreme resistance area of NWMAD (74% control over 16 weeks) was similar to the range of control reported elsewhere with Altosid^®^ XR-treated catch basins over the past 30 years or more.

## 4. Discussion

Altosid^®^ XR (originally Zoecon RF-292) was first labeled for use in 1988 with 1.8% methoprene. In 1990, the methoprene concentration decreased to 1.6% and then increased to 2.1%, a level that has been used in the formulation since 1997. In 1990, Philips et al. [9] observed 62% and 49% control through 90 and 150 days respectively in Altosid^®^ XR-treated catch basins in Fresno, CA, USA. Also in 1990, Knepper et al. [10] reported 70% control in basins over 15 weeks in Saginaw, MI, USA. These studies both evaluated the Altosid^®^ XR formulation with the lowest methoprene concentration (1.6%). Further studies evaluated the current formulation with the highest methoprene concentration (2.1%). Stockwell et al. [11] reported 69% over 19 weeks in Marshfield, WI, USA, in 2005. Mian et al. [12] observed 68% control 4 weeks post-treatment in six catch basins in Riverside, CA, in 2009. Over 2014 and 2015, Staples et al. [13] reported 95% control for the first 70 days post-treatment, >60% at 96 days post-treatment, and essentially 0% at 124 days post-treatment in catch basins in Kalamunda, Australia. More recently in Woodland, CA, Wagner and Wheeler [14] found weekly control in Altosid^®^ XR-treated catch basins to waver between 60% and 80% over 13 weeks. These findings occurred in an area where a range of no to high methoprene resistance in *Cx. pipiens* was observed 2 years prior. Finally, in simulated catch basins, Su [15] found control with these briquets to range from 12.7% to 82.7%, with an average 40.7% over 26 weeks.

Overall, and even with two decades of exclusive methoprene use in catch basins, no loss of control was detected using NWMAD’s operational evaluation methods in areas with resistance categorized as high and extreme. The observed control in all sampled NWMAD areas appear to be within the range of results reported elsewhere, including from over 3 decades and across different regions. When considering these previously reported findings, results of this current work did not align well with expectations of reduced control associated with resistance categories (i.e., moderate, high, extreme resistance). A similar incongruity was noted in Lopez et al. [6]. In that study no loss of control effectiveness was observed with the use of Sumilarv^®^ WSPs (pyriproxyfen) in catch basins despite nearly all sites where that larvicide was applied to have high and extreme pyriproxyfen resistance in *Cx. pipiens*. The methods for identifying such sustained levels control despite reported resistance, however, were not reported.

These operational observations of continued control in areas with high and extreme resistance are counter to many studies that have connected larvicide resistance to control failures in field settings [16,17,18,19,20,21,22,23,24]. However, as a metric, failures may be problematic and this term is often reported without any definition. Designating that treatments have failed can be more subjective as different programs and investigators may use different thresholds and/or definitions for control failure. Of such work, either no definition for failures was provided [16,18,20,21,22,23,24] or failure was defined as when mortality associated with treatments dropped below 80% [17,19]. Furthermore, failure is a binary metric (fail/not fail) that may not capture the range of control that can occur with larvicide applications and may inaccurately suggest that no control at all exists.

There are abundant limitations when considering this work’s data. The methods for data collection were intentionally designed to more quickly inform operations (i.e., to determine how well treatments are working and if re-treatments are needed) and collected in a far less scientifically rigorous manner than those generated with true experimental designs. It could also be that *Cx. pipiens* larvae in the western area of the district were more resistant than previously thought. Although Lopez et al. [6] did not sample the same three western sites where populations with susceptibility and moderate resistance were detected in 2023 and 2024, populations with high resistance were later detected in 2025 as close as approximately 2 mi/3.2 km away from the western clusters of basins of this current work. To date, more larvicide resistance testing effort has been put in the eastern portion of NWMAD. Thus, with more and geographically expanded testing, higher levels of resistance may be found in that western “more methoprene susceptible” area.

It is also unknown how well mosquitoes tested in resistance assays can be broadly representative of other populations from sites nearby or how resistance levels may vary over time. The eastern (more methoprene resistant) and western groups of basins (more methoprene susceptible) may have also been exposed to different environmental and structural factors that could have potentially affected control with the Altosid^®^ briquets. These could include exposure to different amounts of rainfall, different volumes of sump water, and varying levels organic material in sump water. All of these given factors could facilitate flushing out or burying of briquets. An unpublished and non-systematic survey performed by the author from 2016–2026 of 531 catch basins within the north and northwestern suburbs of Chicago found those basins to hold an average of 136.7 ± 4.5 SE gallons of water within sumps with a median of 125.3 gallons (average = 517.5 ± 17 SE liters, median = 474.3 L). The range of observed basin sump water volumes was 0.005–527.8 gallons (0.02–1997.9 L), suggesting wide variability of water volumes held across basins.

Basin density also did vary from the western side (approximately 100–300 catch basins per sq. mi [2.6 sq km]) to the eastern more urban side of the district (approximately 400–600 catch basins per sq. mi [2.6 sq km]). Anecdotally, in the eastern portion of the district, it was more difficult to find basins that held pupae or even standing water to be sampled during the time of inspections. As such, 75 (83.3%) of the 90 basins that contained pupae in the western area of NWMAD at the time of the 2025 inspections were found within the same 2 sq mi (5.2 sq km) area of Elgin, IL. Generally, the sumps in these western basins also appeared to be smaller, holding less water and organic material. The absence of inferential statistical analyses on these observational data and the lack of information on potentially important environmental variables such as rainfall, organic matter, hydrological conditions, make it difficult to determine whether the observed differences in larvicide control are more attributable to methoprene resistance or to other uncontrolled factors.

Although NWMAD’s methods to evaluate larvicide treatments may have been sufficient to differentiate effective control between larvicide formulations and doses, these methods may not be appropriate to detect any loss of effective control due to resistance. If such a limitation is not unique to NWMAD, other mosquito programs may similarly lack the capacity to detect reductions due to larvicide resistance without more resources, guidance and expertise. Thus, although both resistance testing and control evaluations are basic components of IMM, results of these efforts may not always align. This can complicate decisions of larvicide choice.

Given this work’s many limitations and an overall lack of true scientific rigor, it highlights a need to better understand how results of larvicide resistance assays may inform expectations of control in the process of mosquito control operations. Because larvicide control in catch basins may be influenced by a wide number of factors such as constituents in sump water, larvicide formulation, larvicide dose, rainfall, and sump debris, it may also be useful to consider that a range of control should be expected for all larvicides in these habitats. Thus, lower than expected control may not always be driven by any single factor such as resistance. With the different chemistries and mechanisms of larvicide active ingredients it may be that currently used resistance categories (e.g., susceptible, moderate, extreme) may not have the same operational implications for every larvicide formulation, dose, habitat, and mosquito species. Finally, laboratory findings of resistance can indicate that a problem is developing prior to detecting loss of control in operational settings. However, the speed (within a single season to perhaps decades) at which loss of control due to larvicide resistance becomes more easily detectable operationally may likewise vary by active ingredients, formulations (including percent of active ingredient), intensity of larvicide use, and targeted mosquito species.

It remains unknown how effective larviciding programs can be in reducing biting adult populations on a much broader level. In the nearby Milwaukee metropolitan area, a recent study estimated that a 94% reduction in pupae in catch basins from treatments of 20 g of VectoLex^®^ FG (Valent BioSciences LLC, Libertyville, IL, active ingredient, 7.5% *Lysinibacillus sphaericus* [Meyer and Neide 1904], Ahmed et al. 2007) strain ABTS-1743) was associated with a 37% reduction in adult *Culex* spp. mosquitoes captured in nearby gravid traps [25]. While encouraging, such reductions are likely dependent on the density of catch basins and their relative contribution to local adult mosquito populations. In that Milwaukee metropolitan study, the densities of treated catch basins were approximately 640–840 per sq mi (251–326.8 catch basins per km^2^). If the density of treated catch basins is higher in a region, such as commonly found in more urban areas like Milwaukee and Chicago, less effective larvicide treatments may still result in detectable reductions in local adult mosquito populations. Alternatively, if the density of treated catch basins is lower in an area, treatments of these basins will likely make less of an impact. Thus, in areas with lower treated basin densities, even 100% control in these catch basins during an entire mosquito season may not result in any detectable reduction of adult mosquitoes.

Another consideration NWMAD now faces is that resistance levels categorized as “moderate resistance” or higher has been detected in *Cx. pipiens* populations to all major active ingredients (methoprene, *L. sphaericus*, pyriproxyfen, and spinosad) in some sites of the Chicago metropolitan area. Within the NWMAD’s operational area, multiple populations with moderate and high resistance to pyriproxyfen [6] and one population with moderate resistance to spinosad has been reported to the district [26]. For the 2026 season, NWMAD chose to apply 2 WSPs (50 g) of Sumilarv 0.5 G to all basins and expanded treatments from approximately 50,000 to more than 80,000 basins. This decision was driven by results of NWMAD’s operational evaluations and those from the mosquito abatement district adjacent to NWMAD, the North Shore Mosquito Abatement District. Both districts’ larvicide evaluations used the same catch basin sampling methods as described in this current work and were performed by the author. Results of these operational and unpublished larvicide evaluations suggested that (1) the 2 WSP dose was observed operationally to provide the highest level of control for longer than all other non-pyriproxyfen larvicide formulations evaluated by both agencies, and (2) the 2 WSPs dose appeared to provide a similar level of emergence inhibition and duration to that of the 3 WSP dose. The decision to increase the number of treated catch basins from 50,000 to more than 80,000 was believed to increase the chance of reducing local adult mosquito populations to levels that minimize human risk of West Nile virus. Prior to 2026, basins only received larvicide treatments if they held water at the time technicians were out making applications. Currently, all basins now receive a larvicide application no matter if they are dry or hold water at the time of treatments, nearly doubling the number of treated catch basins.

The intent and scope of this work was not to establish that resistance has no effect on observed field effectiveness. Rather, that a disconnect was observed between operational larvicide evaluations in catch basins and expectations of control (or lack thereof) associated with currently used resistance categories (i.e., susceptible, moderate, high, or extreme resistance). In the author’s opinion, it remains unclear how mosquito control programs should weigh currently available evidence from larvicide resistance testing with operational control evaluations. Mosquito control programs using the IMM approach need to consider results of such efforts in a manner that both assures good stewardship of pesticides and the program’s capacity to minimize human risk of mosquito-borne diseases. This lack of understanding of how resistance may influence operational outcomes has been noted elsewhere [27,28]. A simple step forward could be using language in guidance or best practices documents that makes a distinction between what resistance and control evaluations are assessing. For example, more accurately acknowledging the difference between “efficacy” (measuring performance under controlled, laboratory conditions such as commonly used resistance assays) and “effectiveness” (measuring performance in less or uncontrolled “real-world” conditions such as operational control evaluations) could be helpful to decision-makers. Evaluating pesticide efficacy and effectiveness are both core components of IMM and ideally should be performed together. But such a distinction between the two methods could improve the understanding of available evidence of larvicide performance. Another rather straightforward modification would be to move away from reporting operational control as a failure or failing in favor of noting observed control levels and/or ranges of control over time.

Finally, evaluations of effective control should also consider the scope of efforts (i.e., the number of treated standing water sites) and not just how well a given larvicide works within standing water sites. Such a broad assessment could give important insight into the overall effectiveness of larviciding programs. These larger scale evaluations are likely beyond the resources of many mosquito control programs but create an opportunity for collaboration between mosquito control programs and academic or other institutions.

## Data Availability

The original contributions presented in this study are included in the article. Further inquiries can be directed to the corresponding author.
